# Multiple or metastatic clear cell chondrosarcoma: a case report

**DOI:** 10.1186/2045-3329-4-12

**Published:** 2014-09-16

**Authors:** Marco Manfrini, Silvana Fiscina, Alberto Righi, Jorge M Montes, Daniel Vanel

**Affiliations:** 1 Department of Surgery, The Rizzoli Institute, Via di Barbiano 1/1o, 40136 Bologna, Italia; 2 Department of Pathology, The Rizzoli Institute, Via di Barbiano 1/1o, 40136 Bologna, Italia; 3 Department of Research, The Rizzoli Institute, Via di Barbiano 1/1o, 40136 Bologna, Italia

**Keywords:** Clear-cell chondrosarcoma, Synchronous, Allograft, Curettage

## Abstract

We report multiple synchronous clear-cell chondrosarcomas in a 43-year-old patient. The patient had a lesion in the right proximal humerus and in the left femoral condyle. Bone scintigraphy revealed increased uptake in both foci. Pathological analysis confirmed the diagnosis in both locations. In the proximal humerus, wide resection of the tumour was performed with allograft reconstruction of the joint with osteosynthesis. The femoral condyle was treated with curettage, phenolization, and cementation. Over a follow-up of 10 years no recurrence or metastasis was observed.

## Introduction

Clear-cell chondrosarcoma (CCC) was first described by Unni et al. [1] as a variant of conventional chondrosarcoma and is considered as a low-grade malignant bone tumor. There is a male prevalence of 2:1 and the tumor most commonly occurs in the 3rd and 5th decade of life [1,2].

The tumor is most frequently found in the epiphysis of the long bones (humerus and proximal femur). Most authors agree that the management of choice is wide resection. The estimated survival rate at 5 years is 92% [2,3]. Metastases to the lungs or other bones have been reported [1]. In 2006 Corradi et al. [4] presented four patients with aggressive CCC (with early metastasis or multiple tumor location). Case 4 of Corradi’s series is the patient we are presenting here. We currently doubt the aggressive presentation as the patient has not presented with any local tumor recurrence or metastasis during 10 years of follow-up.

Here we report a case of synchronous CCC with a favorable outcome at 10 years.

## Case report

**Figure 1:**
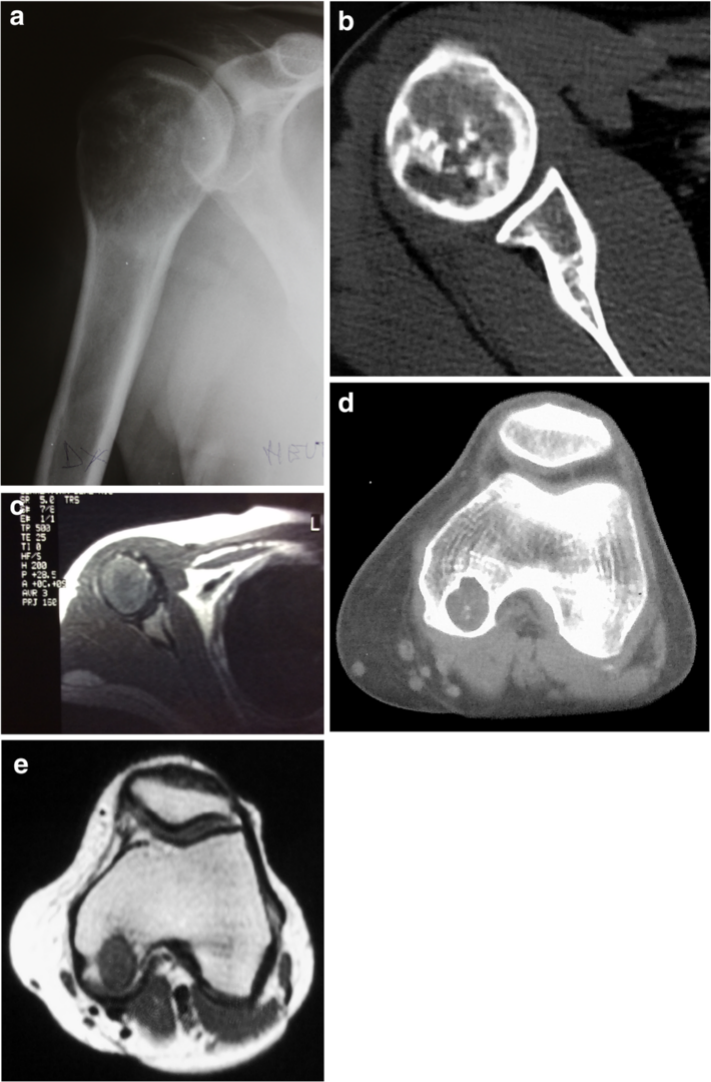
**Humeral tumor. a**: Radiograph of the humerus reveals a lytic proximal lesion with cortical thinning and intralesional calcifications. **b**: CT scan of the shoulder showing intralesional popcorn-like calcifications. **c**: MRI T1 images lesion occupying almost the entire humeral epiphysis, with a preserved cortex and no soft-tissue involvement. **d**: Hematoxylin and eosin staining of clear-cell chondrosarcoma in the humerus consisting of plump cells with well-defined cytoplasmic borders, clear-to-pale eosinophilic cytoplasm and round nuclei. Mitotic figures are scanty and amounts of woven bone are present (x100 magnification). 1**e**: Joint allograft, without signs of recurrence.

**Figure 2:**
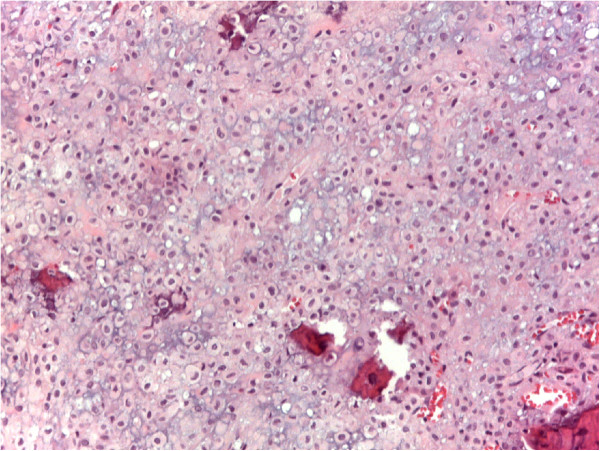
Whole-body bone scan with increased uptake in two foci.

**Figure 3:**
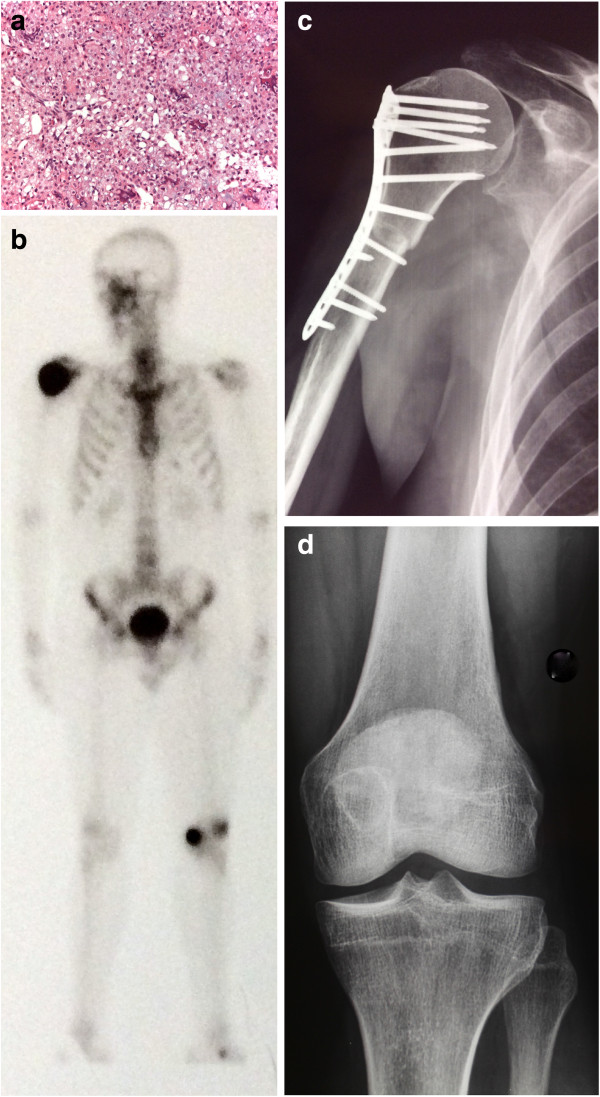
**Imaging of the knee. a**: radiograph: well limited lytic condylar image with a sclerotic rima. **b**: Axial CT scan of the knee showing a lytic lesion with calcifications and without cortical disruption. **c**: MRI T2-weighted image shows a homogeneously hypointense and well delineated lesion. There is no peri lesional edema. **d**: Femur clear cell chondrosarcoma on hematoxylin and eosin staining shows morphologically an indistinct lobularity and fine fibrovascular septa separate sheets of clear cells associated to delicate trabeculae of osteoid and rare multinucleated giant cells (x100 of magnification). **e**: Radiograph of the knee after curettage, phenolization, and cementation.

A 43-year-old male patient was seen with a one-year history of pain in the right shoulder and limited range of motion of the joint. Radiographs revealed a lytic lesion in the proximal epiphyseal humerus, with calcifications, and cortical thinning and expansion of the bone (Figure 1a). Computed tomography (CT) scan of the shoulder revealed a 5-cm-long intracompartimental lesion with cortical thinning and popcorn-like calcifications (Figure 1b). Magnetic resonance imaging (MRI) of the shoulder showed an hypointense on T1- and hyperintense on T2-weighted images lesion occupying almost the entire humeral epiphysis, with a preserved cortex and no soft-tissue involvement (Figure 1c).Bone scintigraphy showed increased uptake in two lesions: in the proximal humerus- already known - and in the contralateral distal femur - an asymptomatic lesion (Figure 2).Radiographs of the knee confirmed a lytic lesion in the medial condyle with a sclerotic margin. The lesion was clinically asymptomatic and the patient had full range of motion in the joint (Figure 3a). CT scan of the knee confirmed a well-circumscribed lytic lesion with a sclerotic rim, cortical thinning without disruption of the bone (Figure 3b) . MRI of the knee displayed a round homogeneous well limited hypodense on T1-weighted sequence lesion (Figure 3c).

CT scan of the thorax was normal and alkaline phosphatase within normal limits.Needle biopsies were performed and the diagnosis of CCC was confirmed in both sites (Figures 1d and 3d).Wide tumor resection of the proximal humerus was performed followed by allograft reconstruction of the joint with osteosynthesis. The femoral condyle lesion was treated with curettage, phenolization, and cementation, in the same time (Figures 1e and 3e). Clinical and imaging follow-up (CT scan of the thorax and radiographs of the knee and shoulder) were performed every six months for three years and yearly thereafter. Over a postoperative follow-up of 10 years, no recurrence or metastasis was found.

## Discussion

CCC is a rare low grade variant accounting for 2% of all chondrosarcomas [1,5,6]. The most frequent symptom is long-term unspecific local pain. Bjorgsson et al. [5] reported that in their series of 47 patients 55% had had a history of symptoms for more than one year, which may be attributed to the slow growth of the tumour [1,6,7]. Although Collins et al. [8] described that tumor located in the proximal humerus was radiologically more aggressive, we have not observed any local recurrence after a follow-up of 10 years. Histological findings showed proliferation of polyhedric clear cells with distinct cytoplasmic borders and central nuclei, giant multinucleated cells, and scattered areas of conventional low-grade chondrosarcoma as well as calcifications within the tumor [8]. These authors suggest that there is a subgroup of CCC with a worse outcome, not predictable by conventional histopathalogical analysis. In these tumours, less expression of certain cell-membrane proteins may contribute to increased clinical aggressiveness. The management of choice is wide tumor resection [1,2,4-6,9,10]. According to Bjorgsson et al. [6] with this treatment modality tumor recurrence rate is 15%. In spite of this recommendation, intralesional resection was chosen in our lesion of the femoral condyle as the lesion was considered non active and was clinically asymptomatic (an incidental finding on whole-body bone scan). Local recurrence after inadequate resection was found to be 83% and 86% [1 and 6, respectively]. In the literature local recurrence at 19 years [11] and metastatic disease at 23 years [2] postoperatively have been described. Although, Donati et al. [5] conclude in their report of 18 cases that alkaline phosphatase may be used in the follow-up as a marker of tumor recurrence and metastasis, in our patient alkaline phosphatase levels were within normal limits both before surgery and during the follow-up period.

The condylar lesion had clinical and imaging features that were similar to epiphyseal chondroblastoma; but the patient was too old, and there was no inflammatory reaction on MR,as usually seen in chondroblastoma. Some authors suggest that CCC may be a malignant chondroblastoma [11,12]. Achim [11] concludes that epiphyseal chondroblastomas occur in younger patients, have a smaller size (from 1 to 4 cm), and are more confined to the epiphysis than CCC.

We found no multiple synchronous or not CCC reported in the literature [4]. Recurrences and bone metastasis during follow-up have been described. Our case has multiple location at the time of diagnosis. Could the condyle lesion be metastatic? Is the humerus the primary location? Or the opposite, which is much more probable, as the humerus lesion is active and the femoral not? On histology, the diagnosis of CCC was definitively confirmed, however, differentiation between a primary and a metastatic lesion is not possible. Immunohistochemically we can make the differential diagnosis, with other pathologies containing clear cells (kidney or lung adenocarcinoma metastastasis), but not between a primary tumor and a metastasis . CCC is positive for S-100 protein (CD 10 -), however, in metastasis, cytokeratins and CD 10 are positive. Karyotyping was not done, as fresh tissue was not planed at the time of the treatment. It could have helped.

Unni et al. [1] reported two patients with multiple bone involvement considered to be metastases and not multiple primary tumors, as visceral metastases were also found.

Unfortunately, in our case previous radiographs of the knee were not available. As in this location the lesion was small, asymptomatic, and was an incidental finding on the bone scintigraphy while physical examination was normal and the imaging findings indicated a non aggressive lesion, it was considered to be an inactive lesion. The fact that the patient had no other metastasis in the follow-up is also in favor of two primary lesions, even if formal proof cannot be established. Due to the slow growth of the tumor, long-term follow-up is essential in CCC, as local recurrence more than 20 years post-operatively has been described.

## Consent

Written informed consent was obtained from the patient (it is the way accepted by our ethicalcommittee for old patients). Approved by the ethical committee of the Rizzoli Institute.

## Competing interests

The authors declare that they have no competing interests.

## Authors’ contributions

MM operated and followed the patient, proposed the subject. SF wrote the article. AR provided and checked the histology. JM checked the article. DV provided the images, and supervised the article. All authors read and approved the final manuscript.

## References

[B1] Unni KK Dahlin DC Beabout JW Sim FH Chondrosarcoma: clear-cell variant. A report of sixteen cases J Bone Joint Surg Am 1976 58 676 683 932066

[B2] Bagley L Kneeland JB Dalinka MK Bullough P Brooks J Unusual behavior of clear cell chondrosarcoma Skeletal Radiol 1993 22 279 282 8316872 10.1007/BF00197674

[B3] Yamaguchi H Isu K Ubayama Y Yamawaki S Gotoh M Miyagawa A Minami A Matsuno T Sasaki T Nojima T Clear cell chondrosarcoma. A report of two cases and review of literature Acta Pathol Jpn 1986 36 1577 1585 3541492 10.1111/j.1440-1827.1986.tb02829.x

[B4] Corradi D Bacchini P Campanini N Bertoni F Aggressive clear cell chondrosarcoma: do distinctive charasteristics exist?: a report of 4 cases Arch Pathol Lab Med 2006 130 1673 1679 17076530 10.5858/2006-130-1673-ACCCDD

[B5] Donati D Yin JQ Colangeli M Colangeli S Bella CD Bacchini P Bertoni F Clear cell chondrosarcoma of bone: long time follow-up of 18 cases Arch Orthop Trauma Surg 2008 128 137 142 10.1007/s00402-007-0353-4 17522879

[B6] Bjornsson J Unni KK Dahlin DC Beabout JW Sim FH Clear cell chondrosarcoma of bone. Observations in 47 cases Am J Surg Pathol 1984 8 223 30 10.1097/00000478-198403000-00009 6703199

[B7] Ayoub K Grimer R Mangham D Carter S Tillman D&R Clear cell chondrosarcoma of bone Sarcoma 1999 3 115 9 10.1080/13577149977749 18521273 PMC2395420

[B8] Collins MS Koyama T Swee RG Inwaeds CY Clear cell chondrosarcoma: radiographic, computed tomographic, and magnetic resonance findings in 34 patients with pathologic correlation Skeletal Radiol 2003 32 687 694 10.1007/s00256-003-0668-3 14530882

[B9] Cannon CP Nelson SD Seeger LL Eckardt JJ Clear cell chondrosarcoma mimicking chondroblastoma in a skeletally immature patient Skeletal Radiol 2002 31 369 372 10.1007/s00256-002-0519-7 12073124

[B10] Le Charpentier Y Forest M Postel M Tomeno B Abelanet R Clear-cell chondrosarcoma: a report of five cases including ultrastructural study Cancer 1979 44 622 629 10.1002/1097-0142(197908)44:2<622::AID-CNCR2820440232>3.0.CO;2-E 476572

[B11] Kaim AH Hugli R Bonel HM Judnt G Chondroblastoma and clear cell chondrosarcoma: radiological and MRI charasteristics with histophatological correlation Skeletal Radiol 2002 31 88 95 10.1007/s00256-001-0450-3 11828329

[B12] Schajowitz F Tumors and tumor-like lesions of bone and joints 1981 New York: Springer-Verlag 195 198

